# An innovative technique for palatal reservoir construction in complete dentures: A case report

**DOI:** 10.15171/joddd.2018.035

**Published:** 2018-09-18

**Authors:** K S Bharanija, V Ashok, Anandapandian Ponsekar Abraham

**Affiliations:** ^1^Department of Prosthodontics, Meenakshi Ammal Dental College & Hospital, Tamil Nadu, India; ^2^Department of Prosthodontics, Saveetha Dental College, Chennai, Tamilnadu, India; ^3^Department of Prosthodontics, Balaji Dental College & Hospital, Tamil Nadu, India

**Keywords:** Artificial saliva, complete dentures, radiation-induced abnormality, xerostomia

## Abstract

The retention and comfort of wearing prosthesis mainly depends on saliva. In reduced saliva conditions, such as xerostomia or radiation therapy, the oral mucosa tends to become dry and ulcerated, leading to the patient's inability to retain the prosthesis. Various techniques have been reported in the literature regarding the use of a reservoir with holes in dentures. The results have not been satisfactory because the flow of the salivary substitute could not be controlled and with frequent cleaning of the denture being necassary to maintain the patency of the reservoir holes. A newer technique for incorporation of a palatal reservoir with controlled artificial salivary flow in complete denture is being explained in this article, which improved the denture retention, comfort, mastication and speech of the patient.

## Introduction

In removable prosthodontics, the prosthesis is but one component of the system, which consists of the denture base, the salivary layer and the oral tissues. Salivary wetting mechanics are necessary to create adhesion, cohesion, surface tension and formation of a vacuum pressure for seating of the dentures, contributing significantly to denture retention.^1^ It also helps protect and cleanse the oral mucosa, reduces the harmful effects of strong acids and bases by its buffering action, regulates water balance and lubricates the oral cavity, making it easy to swallow and speak.^2^

Xerostomia is a clinical condition caused by a decrease in the production of saliva. It might result from certain medications, Sjögren's syndrome, diabetes mellitus, renal failure and radiation therapy. Soreness and ulceration, loss of the denture retention, alteration in taste perception and difficulty in mastication and swallowing are among the problems encountered due to lack of saliva.^2-5^

The depressed salivary action can be reversed through the use of various mechanical or chemical stimulants.^2,6^ The complete absence of salivary flow and salivary functions can be restored to some extent by using salivary substitutes and their frequent oral self-administration by the patient.^2^ To overcome this, various techniques have been described in the literature, including incorporation of reservoirs with holes in maxillary or mandibular dentures or both, to allow the consistent flow of salivary substitute in the oral cavity.^2,5,7-9^ Although they have proved beneficial, the results have not been quite satisfactory because flow of salivary substitute could not be properly controlled, leading to frequent refilling of the reservoir. The food particles also enters the reservoir through the reservoir holes, contaminating the artificial saliva and preventing the flow of salivary substitute into the mouth.^10^

In this article a technique is presented for fabrication of a palatal reservoir designed for maxillary dentures with controlled salivary flow.

## Case report

A male patient aged 68 years referred, complaining of inability to speak and eat properly due to missing teeth. He had been wearing dentures for the past 8 years. He also complained of the inability to use the dentures for the past 6‒8 months due to dryness and a burning sensation of the mouth. He was a known diabetic under medication for the past 24 years. On intraoral examination, he was found to be completely edentulous. The mucosa appeared pink to reddish in many regions and got stuck to the diagnostic instruments due to lack of saliva. Therefore after examination and evaluation of the oral conditions, complete dentures with a palatal reservoir were planned.

### Technique

**Figure 1:**
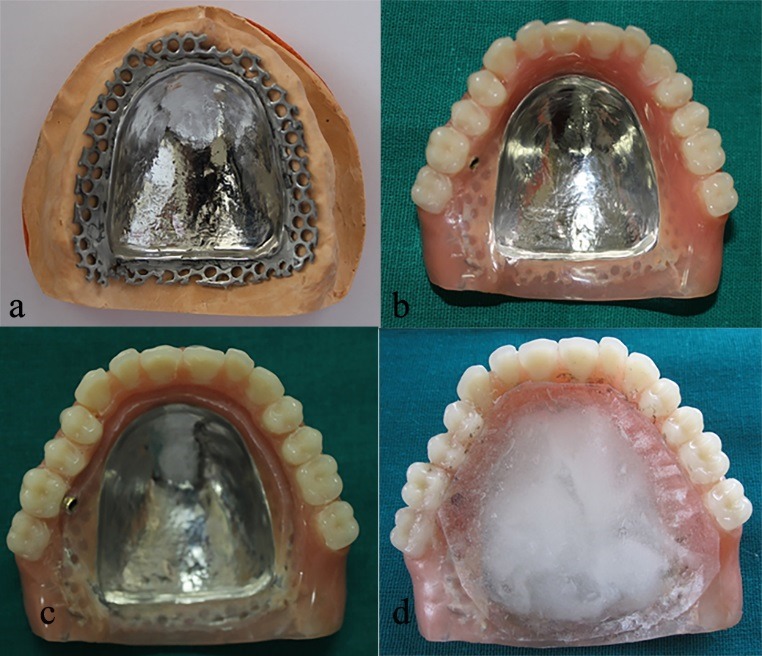
(a)Chrome-cobalt palatal plate fabricated. (b) Processed upper denture with metal inlet tube in the first molar region.Figure (c)Undercut created 2mm above metal and acrylic junction. (d)Functional molding of available palatal space using soft relining material.

**Figure 2:**
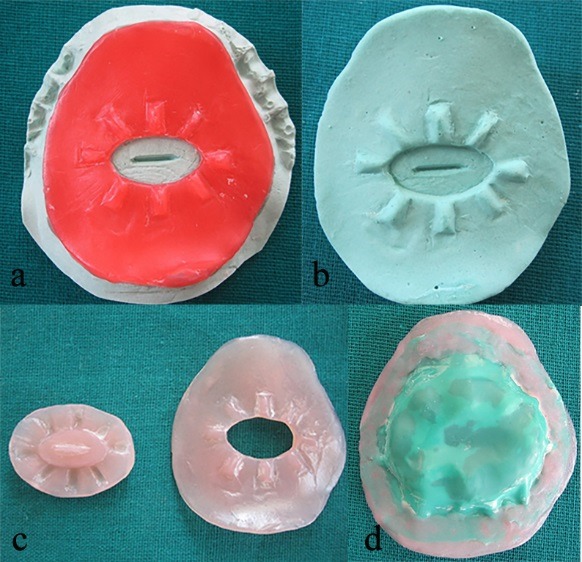
(a)The first wax pattern made on the first plaster index. (b) The second plaster index made by duplication of the first wax pattern. (c)The wax patterns were processed using heat-cured acrylic resin to form acrylic plates. (d)The acrylic plates were held together in place using rubber dam and self-cured acrylic resin.

**Figure 3:**
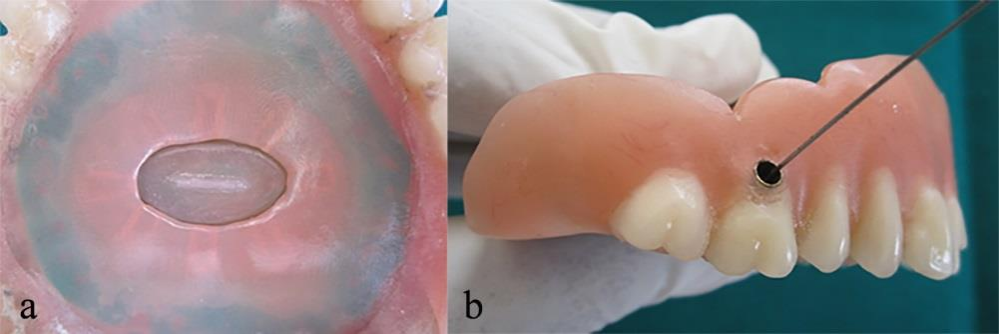
(a)Completed palatal reservoir. (b)The reservoir was filled with artificial saliva.

**Figure 4:**
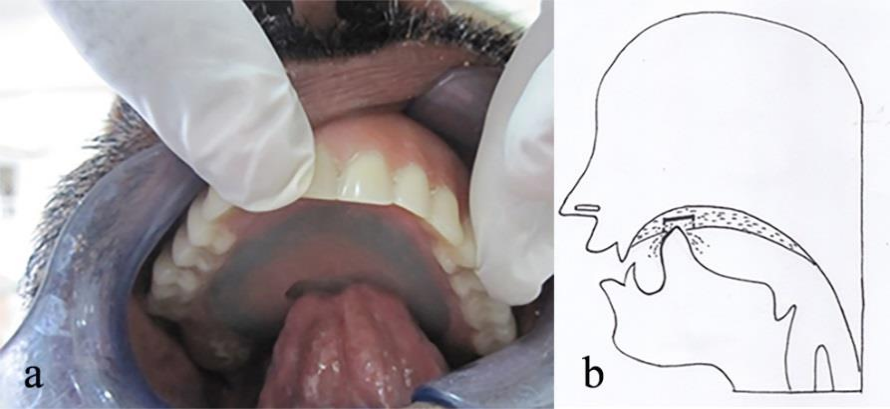
(a) Complete denture with palatal reservoir in function intraorally. (b)Illustration depicting the functioning of palatal reservoir.

1. The complete dentures were constructed until the trial stage in a conventional manner. Cobalt-chromium (Wirobond, Bego Medical GmbH, Germany) inlet tube of about 10 mm in length and 2 mm in diameter was placed underneath the maxillary first molar acrylic teeth region, inclined palatally, passing from the buccal to the palatal surface.
2. A chrome-cobalt complete palatal plate was fabricated, measuring about 0.4 mm in thickness in center and 1 mm at the site joining the acrylic region. It covered the center of the palate and terminated 5 mm anterior to the posterior extension (Figure 1a).
3. The denture was processed and finished with the patency of inlet tube maintained, and the occlusal errors were corrected (Figure 1b).
4. An undercut of 10° was created 2 mm above the junction of the metal palate and acrylic resin, which served as external finish line (Figure 1c).
5. Soft tissue reliner (GC Soft Liner, GC Corporation, Japan) was placed on the polished palatal surface and the patient was asked to wear it for 24 hours to functionally contour the soft tissue liner (Figure 1d).
6. The soft tissue liner extending into the created undercut was removed and a plaster index (first index) (Kaldent, Kalabhai, Mumbai) was made over the contoured soft liner and thin strip of modeling wax (The Hindustan Dental Products, Hyderabad) was adapted over the inner surface of the index (first pattern) extending up to the external finish line.
7. An oval-shaped cut of about 1.5×1 cm in dimension was made in the middle one-third of the first pattern. Elevations were made with wax, along the sides of the cut, measuring 0.5×0.25 cm to serve as orientation ridges. The first plaster index was scraped, corresponding to the center of the cut, to a depth and width of 1 mm, extending for about 8 mm in length (Figure 2a).
8. The pattern, along with the index, was duplicated with alginate (Algitex, DPI, Mumbai) and the second plaster index (Kaldent, Kalabhai, Mumbai) was made (Figure 2b).
9. The second wax pattern was made on the second plaster index, covering the center portion of index extending onto the elevations. The wax patterns were processed with heat-cured acrylic resin (Heat Cure, DPI, Mumbai) (Figure 2c).
10. An adequate piece of rubber dam (Dental Dam, Coltene Whaledent, Germany) was taken to hold the acrylic plates in position, which was then attached to the plates using self-cured acrylic resin (RR Cold Cure, DPI, Mumbai) (Figure 2d).
11. The entire setup was then placed in position, corresponding to the undercut region in the denture and sealed securely with self-cured acrylic resin (RR Cold Cure, DPI, Mumbai). The excess material was trimmed and the denture was finished (Figure 3a). The undercut created prevented the slip of the secured acrylic plates and helped in proper positioning in the denture.
12. The second acrylic plate was slightly pushed up and the holes were made on the sides in the rubber dam with a straight probe. It permitted the flow of artificial saliva into the mouth only when the second acrylic plate was in raised position.
13. The salivary reservoir was filled with artificial saliva (Wet Mouth, ICPA Health products Ltd, Mumbai) by injecting through the inlet tube (Figure 3b) and was closed using a customized rubber stopper.
14. The denture was then delivered to the patient and the stability of the denture was checked. The patient was trained to push up the inner acrylic plate with his tongue (Figures 4a and 4b) with the aid of the horizontal elevation in the second acrylic plate.
15. The patient was periodically reviewed and the number of holes was increased until adequate flow was achieved.
16. Proper instructions were provided for the patients regarding the denture care, as well as how to load, use and maintain the denture reservoir.

## Discussion

Edentulous patients suffering from xerostomia may complain of not only a dry mouth, but also of difficulty in other normal functions. Extreme discomfort in wearing dentures can be very damaging to the dry mouth. It is due to the reduced surface tension due to xerostomia, rendering the denture loose and potentially more traumatic. The buccal mucosa, tongue and lips tend to stick to the denture, making speech and mastication difficult. Retention can be improved by moistening the mouth with a sip of water and then spraying with salivary substitute before placing the denture.^1^

Disadvantage of all artificial saliva samples is their relatively short retention time in the oral cavity. Frequent administration is required for every few hours, which many patients find inconvenient. Therefore slow-release devices such as reservoirs have been developed to circumvent this problem.

During construction of complete dentures a very careful and gentle approach is essential for patients with dry mouth as the mucosa and lips are easily traumatized. Silicone impression materials are best tolerated and are the least traumatic to the mucosa. Zinc oxide eugenol paste will adhere to or burn the mouth and materials such as plaster of paris will adhere to the mucosa and abrade it.^8^

Various techniques have been put forth for the construction of denture reservoirs. The palatal salivary reservoir is best preferred because of larger reservoir size when compared to mandibular reservoir. The fluid and food in the floor of the mouth cause clogging of holes in mandibular reservoir and cleaning is difficult.^2,9^ It also provides flow of saliva to the whole mouth compared to the mandibular reservoir where the flow is restricted only to the floor of the mouth. Xerostomia patients with severely atrophied mandibular ridges might also benefit from maxillary dentures.^2^

The major disadvantage of the palatal reservoir is increased palatal thickness and constricted oral space leading to speech alteration and difficulty in swallowing. This was overcome to some extent by functional molding of the palate using soft liner material. Functional molding of the material helped in recording available tongue space, so that the reservoir can be constructed with no or minimal discomfort.^8^ However, use of palatal reservoir in patients with shallow palatal vault is questionable.

The use of chrome cobalt palate in the maxillary denture reduced the thickness of the denture in the palatal aspect and furthermore created more space for the reservoir. Also the metal denture base exhibited better retention properties and better wetting compared to acrylic resin denture base.^8^

The undercut serving as external finish line, created above the junction of the metal palate and acrylic resin provided mechanical retention for the reservoir plate. The external contour of the inner plate served as a guidance for the patient and for functional use of the reservoir. The outer and inner acrylic plates of the reservoir were held together by the rubber dam, which served as an elastic diaphragm helping in controlled and prolong release of the artificial saliva into the oral cavity. It also prevented the entry of food particles into the reservoir in rest position. Separate loading and release holes helped in better maintenance of the prosthesis.

The average flow of saliva for a normal individual is 500‒1500 mL/day. The salivary denture reservoir will aid in maintaining the moisture in oral cavity. However, frequent intake of water by the patients was recommended.

## Conclusion

A denture with palatal salivary reservoir was constructed for a xerostomia patient; it provided prolonged wetting of the oral structures and also aided in better functioning of mastication, speech and deglutition. Periodic recall and review were carried out and observation was noted. Patients’ motivation and cooperation played an important role in the success of the prosthesis.

## Acknowledgements

None.

## Funding

None.

## Competing Interests

The authors declare that they have no competing interests with regards to the authorship and/or publication of this article.

## Ethics Approval

None.
